# Dynamic loading and kinematics analysis of vertical jump based on different forefoot morphology

**DOI:** 10.1186/s40064-016-3682-3

**Published:** 2016-11-22

**Authors:** Yang Shu, Yan Zhang, Lin Fu, Gusztáv Fekete, Julien S. Baker, Jianshe Li, Yaodong Gu

**Affiliations:** 1 Faculty of Sports Science, Ningbo University, No. 818, Fenghua Road, Jiangbei District, Ningbo, Zhejiang Province China; 2 Research Academy of Grand Health Interdisciplinary, Ningbo University, Ningbo, China; 3 Department of Mechanical Engineering, University of West Hungary, Szombathely, Hungary; 4 Department of Automation, Biomechanics and Mechatronics, The Lodz University of Technology, Lodz, Poland; 5 School of Science and Sport, University of the West of Scotland, Paisley, UK

**Keywords:** Foot morphology, Toes, Vertical jump, Plantar pressure

## Abstract

**Purpose:**

This study examined differences in ankle motion and plantar pressure between habitually barefoot male (HBM) and habitually shod male (HSM) during vertical jump.

**Methods:**

Eighteen habitually barefoot males and twenty habitually shod males volunteered to join the test. Distance between hallux and second toe was measured with Easy-Foot-Scan. Plantar pressure and ankle kinematics were measured with EMED force platform and Vicon motion analysis system respectively. T test was taken to analyse the significant differences using Stata 12.0 software.

**Results:**

The distance between hallux and other toes in HBM was greater than it in HSM. HBM showed larger plantar loading under hallux and medial forefoot, while HSM showed lager plantar loading under medial and central forefoot. HBM had smaller ankle plantarflexion, eversion and external rotation than HSM.

**Conclusion:**

Findings of this study provide basic information for further studies on different hallux/toe function in motion control between habitually shod and barefoot populations.

## Background

Human is bipedal species using two feet to stand and move. Franklin et al. (2015) considered that human feet took the effort of balance and movement control. Morphological differences in foot could cause many foot malfunctions, disorders and deformity (Ledoux et al. 2003). Furthermore, foot morphology had a close relationship with areas: forefoot and toes have been reported to be the prominent target areas (Lambrinudi 1932; Rolian et al. 2009; Hoffmann 1905; D’AoÛt et al. 2009). Wolf et al. (2008) found that acquired behaviour such as footwear wearing may lead to foot structure deformation, such as flatfoot and hallux valgus. Toe separation of habitually barefoot populations showed to be more obvious compared with habitually shod populations (Wolf et al. 2008). In addition, previous studies indicated that habitually barefoot individuals were less likely to be injured than habitually shod ones during running (Robbins and Hanna 1987; Robbins et al. 1988). Lieberman et al. (2010) ascribed this difference to different foot strike patterns. Clinical research presented that metatarsal pathologies were more critical in habitually shod populations than in habitually barefoot populations (Zipfel and Berger 2007).

Jumping as a fundamental motion in sports frequently leads to lower limb injuries, primarily due to the rapid shock to lower limbs at landing (Vint and Hinrichs 1996; Doherty et al. 2014). Ankle sprain has been considered as one of the most common injuries in various sports with frequent jump motion such as volleyball, basketball and soccer. According to the survey, there are approximately 5600 incidences of ankle sprain per day in the UK, a mere between 3 and 5% of all Emergency Department visits (Pijnenburg et al. 2000). Larger plantar loading at forefoot and toes areas in take-off and landing may increase the risk of metatarsal injuries. However, whether there are differences in ankle motion and plantar loading between habitually barefoot populations and habitually shod populations in jumping remained to be unclear.

Therefore, the purpose of the study was to investigate difference in ankle kinematics and plantar pressure under forefoot and toes regions between habitually shod male (HSM) and barefoot male (HBM) during vertical jump based on different forefoot morphology. It was hypothesised that HBM and HSM would present different ankle motions (ankle variation angles and maximal or minimal angles) and plantar pressure characteristics related to different hallux and second toe separation.

## Methods

### Participants

**Fig. 1:**
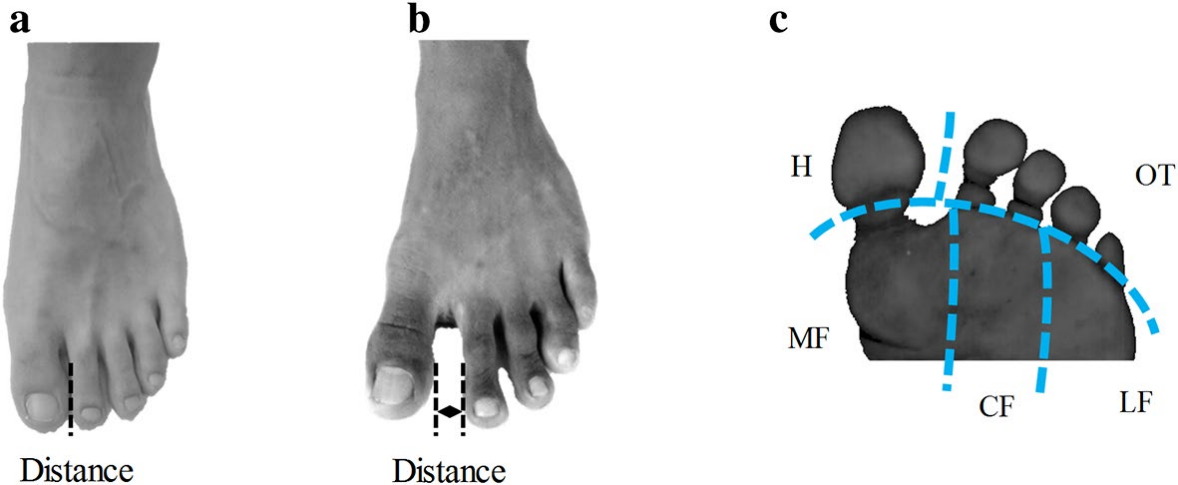
Foot of habitual shod subject (**a**), foot of habitual barefoot subject (**b**) and anatomical parts of plantar pressure (**c**)

Eighteen habitually barefoot males and twenty habitually shod males volunteered to join the test. All participants are Ningbo University students. The HBM come from South India, who are accustomed to walking and exercising barefoot or with slippers/flip-flops since born in daily life. The HSM are accustomed to wearing different kind of shoes since born in daily life. Basic information of participants is listed in Table 1. The Ethics Committee of Ningbo University approved this study (No. 2016FS021) and participants were informed of experiment procedures and requirements with obtained consent. They were free from injury or surgery of their lower extremity in the past six months. Easy-Foot-Scan (EFS), OrthoBaltic (Kaunas, Lithuania) was used to measure forefoot morphological difference of the minimal distance between hallux and the second toes. The minimal distance of HSM was smaller than the distance of HBM (Fig. 1a, b; HSM: 6.28 ± 1.42 mm, HBM: 23.75 ± 2.09 mm, P < 0.001 through the independent-samples T test).

**Table 1:** Descriptive statistics for age, height, mass, and foot length

	Habitually barefoot males (N = 18) Mean (SD)	Habitually shod males (N = 20) Mean (SD)
Age (years)	24 ± 1.2	24 ± 2.1
Height (cm)	165.3 ± 1.2	172.1 ± 1.6
Mass (kg)	65.4 ± 6.9	66.2 ± 6.5
BMI (kg/m^2^)	23.88 ± 0.93	22.31 ± 1.97
Right leg length (cm)^a^	86.5 ± 2.8	89.3 ± 3.9
Right feet length (cm)	25.5 ± 1.4	25.5 ± 0.9

*SD* standard deviation

^a^Right leg length measurement from right anterior superior iliac spine to medial malleolus

### Experiment procedure

**Fig. 2:**
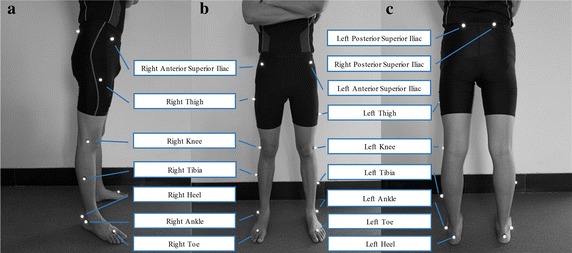
Marker set of three-dimensional kinematic data collection (**a** = side, **b** = front, **c** = rear)

An 8-camera Vicon motion analysis system (Oxford Metrics Ltd., Oxford, UK) was used to collect three-dimensional kinematic data at a frequency of 200 Hz. Participants were required to wear tight shorts. 16 reflective points (diameter 14 mm) were attached on different key locations of right and left lower extremity respectively including anterior–superior iliac spine, posterior–superior iliac spine, lateral mid-thigh, lateral knee, lateral mid-shank, lateral malleolus, second metatarsal head and calcaneus (Fig. 2). Kinetic data were recorded at 50 Hz using an EMED pressure plate (Novel, Germany). All participants were asked to land with forefoot region with right foot on the force plate. The forefoot region was divided into five anatomical parts: medial forefoot (MF), central forefoot (CF), lateral forefoot (LF), hallux (H), other toes (OT) (Fig. 1c). Peak pressure, contact area and pressure–time integral were used to analyse the difference between participants during take-off and landing phase.

Before test, each participant was required to warm up for 5 min. Then participants performed countermovement jump from a suitable pre-squatting motion under barefoot condition. Participants were required to keep their hands on hips in every vertical jump to reduce the energy through the torso activities. Each participant performed five trials, with resting 30 s to avoid fatigue.

Vertical jump height was calculated by the time of flight using Vicon motion analysis system with the formula (Bosco et al. 1983):

$${\text{Jump}}\;{\text{height}}\;\left( {\text{m}} \right) = \frac{{9.80\;{\text{m}}\;{\cdot}\;{\text{s}}^{ - 2} \times {\text{flight time}}\;\left( {\text{s}} \right)^{2} }}{8}$$

Data for analysis were extracted during the taking-off and landing phase. Take-off phase is defined as the period from knee joint starting to flexion to the foot taking off the ground. The instant of take-off is defined as the moment that the vertical ground reaction force closing to 0 N. Landing phase is defined as the period from the foot touching the ground to total knee extension. The instant of landing is defined as the moment that the vertical reaction force higher than 0 N.

### Statistical analysis

All statistical analyses were performed using Stata 12.0 software. The t-test was taken to analysis the significance of jump height, ankle variation range, peak pressure, contact area and pressure–time integral. Significance level P < 0.05 is defined as statistical difference.

## Result

There were no significant differences found in jump height between HBM and HSM (HBM: 0.39 ± 0.11 m; HSM: 0.40 ± 0.13 m, P > 0.05).

**Fig. 3:**
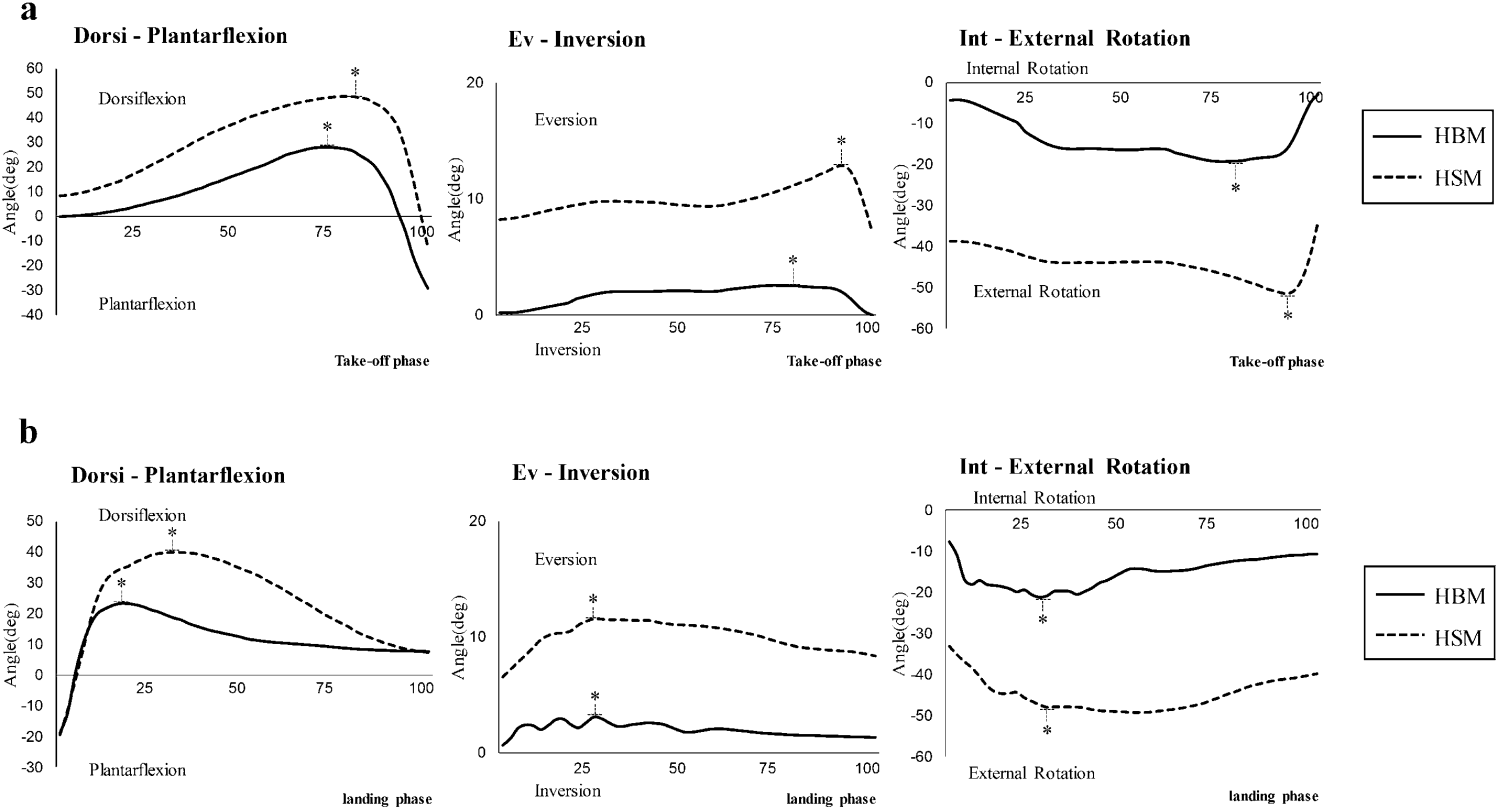
The ankle joints angle curve of the ankle in three planes (sagittal, frontal and horizontal) (**a** = take-off phase, **b** = landing phase, *Asterisk* indicates a statistically significant difference between two groups, P < 0.05)

Ankle joints had significant differences between HBM and HSM during take-off phase (Fig. 3a) and landing phase (Fig. 3b). During take-off phase, ankle of HSM showed significantly larger peak dorsiflexion, eversion and external rotation than HBM. During landing phase, ankle of HSM showed significantly larger peak dorsiflexion, eversion and external rotation than those of HBM.

Table 2 presents comparison of ankle variation angles range during two phases between HSM and HBM. During take-off phase, angle variation range of HBM showed significantly smaller dorsi–plantar flexion and ev-inversion than that of HSM (dorsi–plantar flexion: P < 0.001, ev-inversion: P < 0.001). During landing phase, angle variation ranges of HBM showed significantly smaller dorsi–plantar flexion and ev-inversion angle than that of HSM (dorsi–plantar flexion: P < 0.001, ev-inversion: P < 0.001).

**Table 2:** Comparison of ankle variation range during two phases between HSM and HBM (mean ± SD)

	Take-off phase		Landing phase	
	HBM	HSM	HBM	HSM
Dorsi–plantar flexion	60.85 ± 1.43*	68.25 ± 2.80*	42.45 ± 2.14*	60.40 ± 7.02*
Ev-inversion	3.16 ± 1.49*	6.96 ± 1.49*	3.29 ± 0.34*	6.19 ± 1.49*
gInt-external rotation	20.24 ± 2.47	22.36 ± 6.74	20.00 ± 1.33	18.66 ± 4.67

* Significant different between two groups, P < 0.05

At the moment of take-off, HBM showed significantly larger plantarflexion than that of HSM (P < 0.001). HBM showed to be inversion while HSM showed to be eversion at this moment (P < 0.001). HBM showed significantly smaller external rotation than HSM (P < 0.001). At the moment of landing, HBM showed significantly smaller eversion and external rotation than HSM (eversion: P < 0.001, external rotation: P < 0.001).

**Fig. 4:**
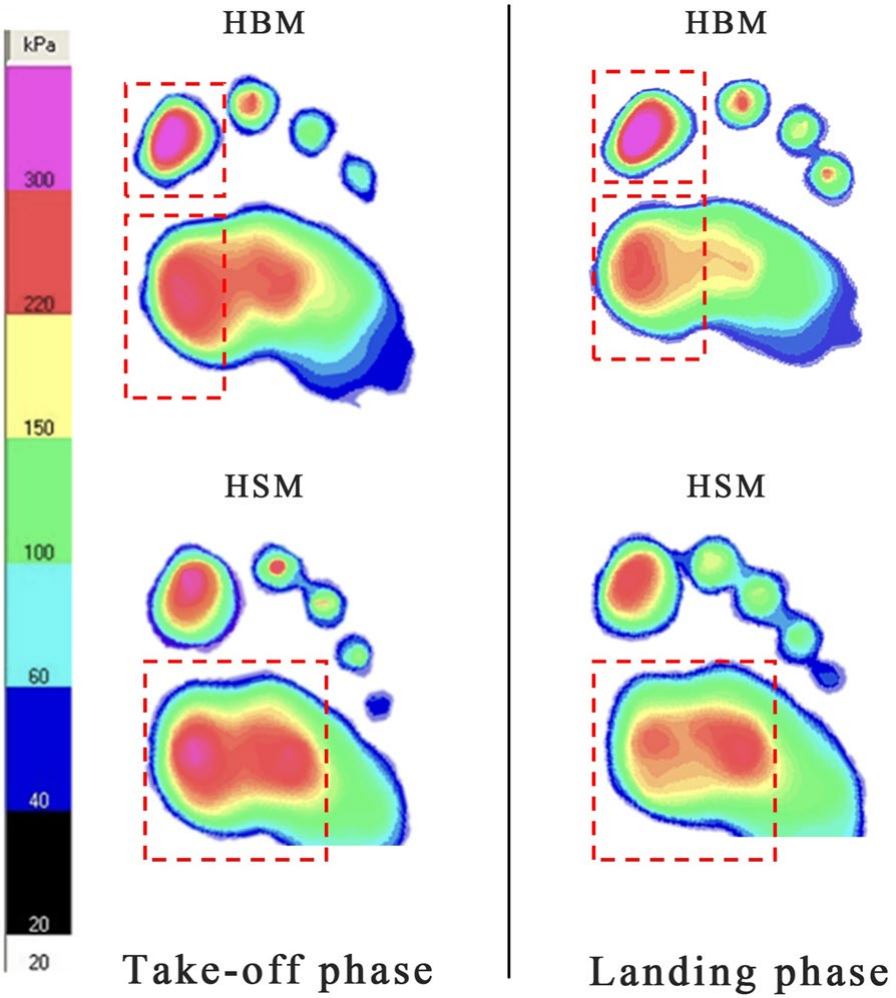
The average peak pressure under forefoot and toes regions during take-off and landing phase. “*Red square*” indicated a significant difference between HSM and HBM

Table 3 and Fig. 4 present comparison of peak pressure, contact area and pressure–time integral between HBM and HSM. During take-off phase, for pressure–time integral, significant differences were found between HBM and HSM in H, MF and CF. HBM showed higher pressure–time integral than HSM in H (P < 0.001). However, the HSM showed greater pressure–time integral than HBM in MF and CF (MF: P = 0.0347; CF: P < 0.001). For peak pressure, significant differences were found in H, MF and CF. HBM showed higher peak pressure than HSM in H (P < 0.001). However, HSM showed higher peak pressure than HBM in MF and CF (MF: P < 0.001; CF: P < 0.001). For contact area, the HBM showed larger contact area than HSM in MF (P = 0.0082).

**Table 3:** Comparison of plantar pressure between HBM and HSM (mean ± SD)

	Take-off phase		Landing phase	
	HBM	HSM	HBM	HSM
Pressure–time integral (Kpa*s)				
H	54.88 ± 15.26*	34.50 ± 11.76*	40.57 ± 7.74*	32.71 ± 11.09*
OT	20.54 ± 5.10	22.70 ± 8.03	19.40 ± 4.41	21.01 ± 3.89
MF	39.36 ± 8.77*	47.75 ± 14.71*	33.50 ± 3.20*	37.07 ± 4.76*
CF	18.59 ± 6.14*	27.66 ± 10.81*	21.47 ± 7.46*	27.43 ± 9.50*
LF	13.02 ± 1.56	13.83 ± 4.53	15.11 ± 1.24*	17.25 ± 4.44*
Peak pressure (Kpa)				
H	649.50 ± 260.09*	267.86 ± 69.11*	391.88 ± 188.61*	287.14 ± 71.07*
OT	248.30 ± 86.50	242.14 ± 37.70	219.38 ± 68.13	207.14 ± 66.16
MF	393.50 ± 135.44*	552.14 ± 241.49*	295.63 ± 123.74	295.00 ± 84.23
CF	138.50 ± 71.36*	242.14 ± 124.66*	138.13 ± 53.76*	221.43 ± 88.43*
LF	85.00 ± 44.32	102.86 ± 52.83	101.88 ± 35.19*	128.58 ± 54.75*
Contact area (cm^2^)				
H	8.88 ± 1.67	8.40 ± 0.80	7.53 ± 2.04	7.02 ± 2.05
OT	8.57 ± 1.80	8.69 ± 1.43	6.94 ± 1.67*	5.57 ± 0.46*
MF	13.98 ± 1.72*	12.45 ± 1.75*	13.29 ± 1.70	12.83 ± 1.50
CF	15.58 ± 2.79	14.13 ± 5.95	17.25 ± 2.37*	20.87 ± 2.14*
LF	8.79 ± 3.49	7.43 ± 3.89	11.43 ± 3.33	11.13 ± 1.15

* Significant different between two groups, P < 0.05

During landing phase, for pressure–time integral, significant differences were found between HBM and HSM in H, MF, CF and LF. HBM showed higher pressure–time integral than HSM in H (P = 0.0132), while HSM showed higher pressure–time integral than HBM in MF, CF and LF (MF: P = 0.0083; CF: P = 0.0335; LF: P = 0.0447). For peak pressure, significant differences were found in H, CF and LF. HBM showed higher peak pressure than HSM in H (P = 0.0075). HSM showed higher peak pressure than HBM in CF and LF (CF: P < 0.001; LF: P = 0.0256). For contact area, significant differences were found in OT and CF. HBM showed larger contact area than HSM in OT (P = 0.0011). HSM showed larger contact area than HBM in CF (CF: P < 0.001).

## Discussion

Previously published researches have proved forefoot morphological difference between HBM from India and HSM from China that HBM have more obvious hallux and the second toe separation compared with HSM (Shu et al. 2015; Mei et al. 2015). This study verified differences in ankle kinematics and plantar loading between the two populations in vertical jump. HBM presented significantly larger plantar loading than HSM under hallux, which may be associated with the fact that hallux of HBM was significantly separate from other toes (Ashizawa et al. 1997). Differences in ankle motions also showed significance between HBM and HSM. However, no significant difference in jump height between two groups was observed.

During take-off phase, significant differences in pressure–time integral and peak pressure between HBM and HSM were under plantar regions of H, MF and CF. During landing phase, differences existed under hallux and forefoot. In this case, it further concluded that plantar loading of HBM was large under H and MF, while the pressure of HSM was large under MF and CF. Previous findings in relation to barefoot running suggested that HBM have distinctive features in push-off phase, which may be caused by the more separated toes of this population that could expand and firm the supporting base in gripping (Hoffmann 1905; Wolf et al. 2008; Ku et al. 2012). Since HBM used hallux while HSM used forefoot primarily during take-off, the significantly larger plantarflexion of HBM than HSM could be explained partly. Similarly, the larger ankle variation range of ev-inversion and int-external rotation of HSM conformed to kinetic results that peak pressure of HSM tended to shift laterally compared with HBM. Moreover, Salinero et al. stated that although increased ankle dorsiflexion could affect muscle activation, it would not improve jump performance (Salinero et al. 2014). This is consistent with the result in this study that HBM and HSM showed comparable jump height with different ankle position in the sagittal plane.

During landing phase, HBM showed larger plantarflexion but smaller eversion and external rotation than HSM. These were in line with the kinetic results that HBM showed larger peak pressure under hallux while smaller pressure under central and lateral forefoot. This suggested different functions of the hallux in motion control between HBM and HSM. Mei et al. (2015) also reported larger loading under the hallux among HBM during running, which may reduce impact force to forefoot area.

Ankle sprain is a common lower limb injury in sports, especially during landing phase in jump. Foot rotation has been reported as a principal factor for ankle sprain in clinical literature (Hopkinson et al. 1990). Previous studies have demonstrated that ankle injuries are associated with combined ankle motions of dorsiflexion, eversion and external rotation (Williams et al. 2007; Taylor and Bassett 1993; Wolfe et al. 2001). In this study, HBM showed smaller eversion and external rotation than HSM, indicating that HBM are at lower risk of ankle sprain compare with HSM (Rolian et al. 2009; Robbins and Hanna 1987). On the other hand, Novacheck (1998) and Tam et al. (2014) stated that excessive loading under metatarsal heads would lead to forefoot injuries such as metatarsal fracture. The larger peak pressure under metatarsal heads areas (MF, CF and LF) of HSM observed in this study indicated a higher risk of forefoot injuries among this population.

## Conclusion

HBM and HSM showed different ankle motions and plantar loading in vertical jump, which is potentially due to forefoot morphological difference in the distance between hallux and the second toe. HBM showed larger ankle plantarflexion with smaller eversion and external rotation compared with HSM. Additionally, HBM showed larger plantar loading under hallux and medial forefoot, while HSM showed larger plantar loading under medial and central forefoot. Findings of this study provide basic information for further studies on different hallux/toe function in motion control between habitually shod and barefoot populations.
